# Evaluation of the Risk of Clinical Deterioration among Inpatients with COVID-19

**DOI:** 10.1155/2021/6689669

**Published:** 2021-06-25

**Authors:** Víctor O. Costa, Eveline M. Nicolini, Bruna M. A. da Costa, Fabrício M. Teixeira, Júlia P. Ferreira, Marcos A. Moura, Jorge Montessi, Rogério L. Campos, Andrea N. Guaraldo, Patrícia M. Costa

**Affiliations:** ^1^Medicine, Faculdade de Ciências Médicas e da Saúde de Juiz de Fora–SUPREMA, Juiz de Fora 36033-003, Brazil; ^2^Thoracic Surgery, Hospital Monte Sinai, Juiz de Fora 36033-318, Brazil; ^3^Nursing, Faculdade de Ciências Médicas e da Saúde de Juiz de Fora–Suprema, Juiz de Fora 36033-003, Brazil; ^4^Infectology, Faculdade de Ciências Médicas e da Saúde de Juiz de Fora-Suprema, Juiz de Fora 36033-003, Brazil; ^5^Psychiatrist and Emergency, Hospital Monte Sinai, Juiz de Fora 36033-318, Brazil; ^6^Intensive Therapy, Hospital Monte Sinai, Juiz de Fora 36033-318, Brazil; ^7^Intensive Therapy and Dermatology Hospital Monte Sinai, Juiz de Fora 36033-318, Brazil

## Abstract

This study aims to assess the risk of severe forms of COVID-19, based on clinical, laboratory, and imaging markers in patients initially admitted to the ward. This is a retrospective observational study, with data from electronic medical records of inpatients, with laboratory confirmation of COVID-19, between March and September 2020, in a hospital from Juiz de Fora-MG, Brazil. Participants (*n* = 74) were separated into two groups by clinical evolution: those who remained in the ward and those who progressed to the ICU. Mann–Whitney *U* test was taken for continuous variables and the chi-square test or Fisher's exact test for categorical variables. Comparing the proposed groups, lower values of lymphocytes (*p *=* *<0.001) and increases in serum creatinine (*p* = 0.009), LDH (*p* = 0.057), troponin (*p* = 0.018), IL-6 (*p* = 0.053), complement C4 (*p* = 0.040), and CRP (*p* = 0.053) showed significant differences or statistical tendency for clinical deterioration. The average age of the groups was 47.9 ± 16.5 and 66.5 ± 7.3 years (*p* = 0.001). Hypertension (*p* = 0.064), heart disease (*p* = 0.048), and COPD (*p* = 0.039) were more linked to ICU admission, as well as the presence of tachypnea on admission (*p* = 0.051). Ground-glass involvement >25% of the lung parenchyma or pleural effusion on chest CT showed association with evolution to ICU (*p* = 0.027), as well as bilateral opacifications (*p* = 0.030) when compared to unilateral ones. Laboratory, clinical, and imaging markers may have significant relation with worse outcomes and the need for intensive treatment, being helpful as predictive factors.

## 1. Introduction

First detected in December 2019, COVID-19 caused by the new coronavirus (SARS-CoV-2) has been considered a pandemic by the World Health Organization (WHO) [1]. According to data from the Ministry of Health (MS), of September 2020, 4,717,991 cases have already been detected in Brazil, of which 141,406 have died, resulting in a lethality rate of 3% [2]. On a global scale, the country is the third with the highest number of accumulated cases and the second in the number of deaths, only behind the United States [3].

COVID-19 can be clinically manifested in three ways, 80% of patients are asymptomatic or oligosymptomatic with mild symptoms without complications (fever, dry cough, fatigue, without pneumonia, or with mild pneumonia), 15% progress to hospitalization with moderate symptoms and need for oxygen therapy (tachypnea, drop in oxygen saturation in room air, and signs of respiratory distress), and 5% have the severe form (severe acute respiratory insufficiency (ARDS), septic shock, and multiple organ dysfunction) with an indication for treatment in the intensive care unit (ICU) [1, 4].

In the group of patients requiring hospitalization or ICU, studies suggest possible predictive factors for worse clinical evolution, which would indicate a higher risk of developing ARDS and/or death [5–7]. Up to the Epidemiological Week Bulletin 38, of September 2020, more than 730,000 cases of ARDS hospitalized in Brazil were reported, 53.2% by COVID-19, with a lethality rate of 34.4% [8]. These data corroborate to emphasize the importance of establishing clinical, laboratory, and imaging predictors for a worse prognosis in order to assist in the early recognition of patients at risk.

Thus, the present study aims to assess the risk of severe forms of COVID-19, based on clinical signs, laboratory tests, and computed tomography of patients who were initially in the hospitalization unit.

## 2. Methods

### 2.1. Design, Ethical Aspects, and Procedures

This is a retrospective observational study. The data were collected by the researchers through electronic medical records of patients who were conducted at the Institute of Clinics and Surgeries of Juiz de Fora—Monte Sinai Hospital in the city of Juiz de Fora, Minas Gerais, Brazil. Such hospital has regional coverage in the Zona da Mata of Minas Gerais, with a population of approximately two million inhabitants. The collection was made between March 2020 and August 2020. The research stages began only after approval by the Research Ethics Committee (Opinion no. 4,080,157, Santa Casa de Misericórdia de Juiz de Fora/MG). After collecting information from electronic medical records, data were tabulated using Google Forms. After correction of typing errors, data analysis was conducted.

### 2.2. Participants

The study included all patients who were admitted to the ward sector with symptoms of COVID-19. Inclusion criteria were as follows: patients of any age, nonpregnant, with COVID-19, confirmed by specific laboratory test—RT-PCR. Exclusion criteria were as follows: patients who were not positive for the RT-PCR laboratory exam for COVID-19. In addition, patients whose medical reports were not clear by inappropriate filling were excluded from the data. In the end, 74 participants were included in the study. No patient who participated in the research had received vaccine for COVID-19, given the time when the research was carried out.

For admission to the ICU, the criteria were as follows: acute respiratory failure (no improvement in oxygen saturation, despite the O_2_ supply (Sat O_2_ <93% with 6L/min supply); acute respiratory failure with the need for invasive mechanical ventilation (need for FiO_2_ > 50% for SpO_2_ mentor> 94% or a respiratory rate less than or equal to 24, PaCO_2_> or equal to 50 mmHg and pH < or equal to 7.35, and PaO_2_/FiO_2_ ratio < 200); sepsis or septic shock with hypotension (SBP <90 or MAP <65) and/or signs of tissue hypoperfusion (elevated lactate); and acute organ dysfunctions (acute renal failure, altered level of consciousness, liver failure, etc.)

All patients who participated in the study were initially admitted to the ward, where laboratory tests were performed within the first 72 hours. Depending on the clinical evolution, patients were separated into two groups: those who remained in the ward and those who progressed to the ICU. It is important to note that laboratory tests were performed in the first 72 hours in order to verify predictive factors for admission to the ICU, and the same number of tests was not performed later.

### 2.3. Data Analysis

Continuous variables were expressed as means ± SDs, while categorical variables were summarized as numbers (%). Differences between the characteristics of the result groups were assessed using the Mann–Whitney *U* test for continuous variables and chi-square tests or Fisher's exact test for categorical variables. *p* < 0.05 was considered statistically significant. All data were analyzed using SPSS (20.0.0 IBM SPSS).

## 3. Results

The general demographic and epidemiological characteristics of all patients are summarized in Table 1. The individuals who had hospitalization and did not progress to the ICU were compared with those who progressed to the ICU, in order to assess criteria that can be observed, verifying the evolution of the patient to the ICU. The number of patients who did not progress to the ICU was 59, while 15 progressed to the ICU, six of whom died. The average age among patients admitted to the ward was 47.9 (±SD: 16 5) and 66.5 among patients who progressed to the ICU (±SD: 17 3), with a significant difference in relation to the patient's age (*p* = 0.001), showing that older age qualifies the patient to be admitted to the ICU. There was no statistically significant difference regarding the ethnic group (*p* = 0.696), being a health professional *p* = 0.276), having received the flu vaccine in the last campaign (*p* = 0.488), or having had contact with someone who is sick, that is, individuals with the flu syndrome (*p* = 0.741). In addition, the number of patients who were not residents of the city Juiz de Fora, Minas Gerais, Brazil, proved to be significant with six (8.1%), with a trend of difference between groups (*p* = 0.077). Regarding comorbidities, hypertension (*p* = 0.064), heart disease (*p* = 0.048), and COPD (*p* = 0.039) were more linked to ICU admission. The number of patients with COPD was few, but they were shown to have statistical relevance. As far as hypertension is concerned, it tends to show statistical differences between the groups.

Regarding the symptoms that the patient reported feeling before hospital admission, the presence of odynophagia was a minor factor in patients who will progress to the ICU (*p* = 0.016) since none (0.0%) reported to have presented with such symptoms on admission. Tachypnea at the time of care at the hospital, on the other hand, presented a significant link with future ICU admission (*p* = 0.051).

With regard to radiographic parameters, a statistical comparison was made between patients at the time of admission and at the time of discharge, in order to verify the change in the radiographic pattern as a discharge criterion. Inpatients underwent chest computed tomography (CT) at the time of admission to the hospital and at discharge. The number of patients with ground-glass involvement> 50% is four (6.9%) on admission and 16 (27.6%) with involvement between 25 and 50% of the lung. At discharge, in the analyzed patients, 12 (29.2%) had an occupation of 25–50% of the lungs and nine (21.9%) had an impairment > 50% (Table 2).

Regarding laboratory evaluation, patients were analyzed in various parameters, comparing laboratory tests of hospitalized patients who did not progress to the ICU with laboratory tests of those who progressed to the ICU. In the hospital internment group, there are only patients who were admitted and did not go to the ICU. All examinations were performed until the third day of hospitalization, in order to assess the patient's prognosis.

Comparing individuals admitted to the ICU with those not admitted, it was evident that the lowest lymphocyte count (768.4 ± 340.4; *p* = <0.001), the highest serum creatinine value (1.73 ± 1.68; *p* = 0.009), and higher values of lactate dehydrogenase (LDH) (730.3 ± 307.6; *p* = 0.057), troponin (18.7 ± 26.9; *p* = 0.018), interleukin-6 (45.1 ± 32.2; *p* = 0.053), C4 complement (52.4 ± 12.6; *p* = 0.040), and C-reactive protein (CRP) (102.6 ± 93.7; *p* = 0.053) showed significant differences or statistical tendency when we compared individuals who progressed to the ICU with those who did not (Table 3).

Finally, a comparison of medication usage and radiographic condition at admission was made with the prediction of the patient to progress to the ICU. Regarding the medications used, they were compared in order to verify whether their early use would have an influence on the patient's evolution to the ICU (Table 4). These medications refer to medications that patients used before hospitalization. It was found that most patients using ivermectin 11 (18.9%) progressed to the ICU (*p* = 0.056). On the other hand, with the involvement of ground glass in greater extent or the presence of pleural effusion with the other findings on CT, there is a positive association for the patient's evolution to the ICU (*p* = 0.027), as well as the comparison between those who presented bilateral opacifications with those who had unilateral opacifications (*p* = 0.030). The number of hospitalization days in patients who did not progress to the ICU was 6.2 ± 2.7 and in those who progressed to the ICU was 10.7 ± 8.7 and notably showed a difference in the comparison between the groups (*p* = <0.001). It is important to note that the number of days of hospitalization addresses every day that the patient was in the hospital, including the infirmary and then his evolution to the ICU.

## 4. Discussion

The evaluation of patients admitted to hospital with COVID-19, confirmed by diagnostic tests, allows the prediction of risk factors associated with a worse prognosis and consequent evolution to ICU and/or death. Early recognition of high-risk patients can guarantee intensive clinical care directed by the multidisciplinary team in order to monitor, avoid, and control possible complications, such as ARDS, organ dysfunctions, and the need for mechanical ventilation [9, 10].

Well-established independent predictors, such as advanced age (66.5 ± 17.3; *p* = 0.001), underlying heart disease, and COPD were also found in our study when we compared hospitalized patients who did not progress with those who progressed to the ICU and/or death [5, 6]. Due to the reduced *n* of the research, some data, also defined in the literature, showed only a tendency towards statistical significance for a poor prognosis, such as hypertension (*p* = 0.064) and tachypnea on admission (*p* = 0.051) [5–7].

However, unlike the studies by Minotti et al. [11], Roncon et al. [12], and Zhang et al. [13], our research did not define a statistical relationship or even a trend between male gender, white ethnicity, type 2 diabetes mellitus (DM2), asthma, and immunosuppression with worse clinical prognosis. There was also no significant difference in relation to the prognosis when comparing the groups of hospitalized patients who progressed and those who did not progress to ICU and/or death, with other clinical symptoms such as odynophagia, tachycardia, and fever. Again, it could be owing to the small *n* of our research but could also demonstrate how nonspecific signals/symptoms such as odynophagia and fever are calling attention to a possible inadequacy of the use of these predictors for screening, for example.

Despite all the scientific limits of a small study, when relevant results are shown and can be easily ratified by similar findings in the literature, we must consider these parameters—advanced age, underlying heart disease, COPD, hypertension, and tachypnea on admission—for the screening of high-risk hospitalized patients in order to try to avoid ICU admission rates even more highly than we are seeing [14–16]. Another fundamental point in the development of a clinical score is the possibility of accurately managing patients uniformly identified in different medical settings.

Evaluating the link between the use of several medications—ivermectin, hydroxychloroquine, ceftriaxone, azithromycin, chlorpromazine, enoxaparin—and a better clinical evolution, no statistical prognostic value was found in accordance with previous studies [7, 17, 18]. Instead, a strong relationship (*p* = 0.056) between patients who used ivermectin—an antiparasitic drug that has shown partial antiviral properties in high-dose in vitro studies—and evolved poorly when compared to those who took the medication and did not worsen clinically was noteworthy. It should be noted, over again, that such results may be justified by the bias inherent in the research design and by the widespread misuse of this medication in our setting, which has its adverse effects well described and its effectiveness for COVID-19 indeterminate by large clinical trials, boosting its risks rather than its benefits [19, 20].

In relation to laboratory analysis, among the different tests requested from patients admitted to our service, lymphopenia (768.4 ± 340.4; *p* < 0.001), elevation in Cr (1.73 ± 1.68; *p* = 0.009), increase in troponin (18.7 ± 26.9; *p* = 0.018), and increase in complement C4 (52.4 ± 12.6; *p* = 0.04) were shown to be statistically significant as predictors of a worse prognosis. A trend towards significance was found with an increase in LDH (730.3 ± 307.6; *p* = 0.057), elevated IL-6 (45.1 ± 32.2. *p* = 0.053), and increased CRP (102.6 ± 93.7. *p* = 0.053). Such results comply with Hou et al. [21], Liu et al. [22], and Zhang et al. [23] proposed in their studies. Differently, serum albumin, D-dimer, ferritin, proBNP, and absolute CD8 + cell count were not relevant in predicting the evolution to ICU and/or death [24, 25].

As for image evaluation, our data showed that inpatients with extensive opacifications (>25%), regardless of laterality or the presence of associated pleural effusion, have a worse prognosis (*p* = 0.027). However, when comparing unilateral ground-glass involvement with the bilateral pattern, it proved to be more statistically related to the evolution to ICU and/or death than that (*p* = 0.03) [26, 27]. It is interesting to highlight that, until the moment of this analysis, patients admitted with opacifications to chest CT at admission and who were later discharged from the hospital still had pulmonary involvement on the image despite evident clinical improvement [28]. The length of staying of those who progressed to the ICU (10.7 ± 8.7 days) was significantly longer (*p* < 0.001) than those who did not progress with clinical worsening (6.2 ± 2.7 days) [29].

## 5. Conclusions

Thus, the study in question is established as a pilot project for the creation of scales that can predict the evolution of a patient in an inpatient unit to the ICU. It is evident that the presence of some laboratory markers, clinical criteria, and findings in imaging studies as elucidated in the study may have a significant relationship with the patient's evolution to the ICU.

Therefore, controlled studies with a larger number of patients are necessary in order to establish probable criteria that assess the patient's prognosis in view of the importance of more evidence covering COVID-19 infection given the current situation of the global pandemic.

## Figures and Tables

**Table 1:** Demographic data of the studied sample.

	Place of hospitalization		*p* value
	Hosp_Intern (*n* = 59)	ICU (*n* = 15)	
*Descriptive data*			
Age	47.9 (±SD:16.5)	66.5 (±SD: 17.3)	0.001
Sex (male)	35 (47.3)	12 (16.2)	0.137
White ethnicity	40 (70.1)	9 (15.7)	0.696
Patient is a healthcare professional (yes)	13 (18.0)	1 (1.3)	0.276
Patient had contact with grippal syndrome (yes)	24 (32.4)	4 (5.4)	0.741
Received flu vaccine during the last campaign (yes)	21 (42.8)	6 (12.2)	0.488
Not being a resident of the city (Juiz de Fora)	10 (13.5)	6 (8.1)	0.077

*Comorbidities*			
Hypertension	20 (27.0)	9 (12.2)	0.064
Diabetes	4 (5.4)	0 (0.0)	0.300
Dyslipidemia	0 (0.0)	1 (1.4)	0.203
Immunosuppression	2 (2.7)	0 (0.0)	0.470
Cardiopathy	4 (5.4)	4 (5.4)	0.048
Asthma	1 (1.4)	0 (0.0)	1.000
COPD	0 (0.0)	2 (2.7)	0.039

*Symptoms on admission*			
Tachypnea	45 (62.5)	7 (9.7)	0.051
Tachycardia	12 (16.6)	3 (4.1)	0.929
Cough	47 (63.5)	9 (12.2)	0.175
Dyspnea	21 (28.4)	6 (8.1)	0.752
Odynophagia	17 (23.0)	0 (0.0)	0.016
Sputum production	1 (1.4)	1 (1.4)	0.367
Headache	13 (17.6)	2 (2.7)	0.791
Fever	44 (59.5)	10 (13.5)	0.531
Rhinorrhea	14 (18.9)	2 (2.7)	0.499
Diarrhea	13 (17.6)	3 (4.1)	0.864

SD: standard deviation; *n* (%); *p* values were calculated by the Mann–Whitney *U* test, *χ*2 test, or Fisher's exact test.

**Table 2:** Chest CT results.

	Admission (*n* = 73)			
	*n* (%)	Involvement < 25%	Involvement 25–50%	Involvement > 50%
Unilateral ground-glass opacity	8 (11.0)	8 (100.0)	0 (0.0)	0 (0.0)
Bilateral ground-glass opacity	58 (79.5)	36 (62.1)	16 (27.6)	4 (6.9)

Discharge (*n* = 49)				
Unilateral ground-glass opacity	4 (8.1)	4 (100.0)	0	0
Bilateral ground-glass opacity	41 (83.6)	20 (48.7)	12 (29.2)	9 (21.9)

	Admission		Discharge	
Clear	5 (6.8)		2 (4.0)	
Inflammatory nodules	14 (19.2)		11 (22.4)	
Pleural effusion	3 (4.1)		3 (6.1)	

**Table 3:** Laboratory tests.

	Total	Hospital internment (±SD)	Total	ICU admission after hospital internment (±SD)	*p* value
Lymphocyte count	59	1607 (686.8)	15	768.4 (340.4)	<0.001
Serum creatinine	57	0.88 (0.27)	15	1.73 (1.68)	0.009
Troponin	38	10.9 (48.3)	11	18.7 (26.9)	0.018
Complement C4	47	44.2 (11.5)	9	52.4 (12.6)	0.040
Interleukin-6	18	19.09 (22.0)	4	45.1 (32.2)	0.053
CPR	59	50.8 (61.3)	13	102.6 (93.7)	0.007
LDH	53	541.1 (199.1)	11	730.3 (307.6)	0.057
WBC count	59	6620.8 (3236.8)	15	5905.3 (1586.0)	0.804
Creatine kinase	51	120.3 (188.7)	9	180.1 (170.7)	0.272
Ferritin	48	559.3 (540.2)	12	699.0 (464.4)	0.244
Bilirubin	53	0.54 (0.49)	11	0.47 (0.15)	0.978
NT-proBNP	3	245.5 (341.7)	3	899.9 (986.9)	0.400
Aspartate transaminase	54	41.3 (29.4)	14	38.3 (14.4)	0.710
Alanine transaminase	54	47.9 (34.9)	14	44.4 (40.7)	0.544
Lactate	6	10.6 (1.8)	1	—	0.286
D-dimer	43	0.47 (0.41)	13	0.20 (0.99)	0.435
Ammonia	46	33.6 (16.2)	8	39.6 (14.7)	0.233
Vitamin D	49	27.8 (12.6)	9	24.8 (7.7)	0.822
Complement C3	47	150.8 (37.3)	9	139.8 (24.5)	0.299
Complement CH50	39	203.0 (57.2)	9	191.5 (49.4)	0.716
Immunoglobulin E	46	115.8 (150.8)	9	124.3 (109.9)	0.467
Immunoglobulin A	47	253.7 (105.0)	9	216.0 (84.2)	0.284
Immunoglobulin M	47	122.0 (71.8)	9	107.1 (73.6)	0.366
Immunoglobulin G	47	1107.3 (369.8)	9	989.5 (210.5)	0.337
Triglycerides	44	145.6 (90.6)	8	132.7 (62.4)	0.852
Total cholesterol	43	158.4 (39.5)	8	137.1 (31.8)	0.112
Neutrophils	59	4198.5 (2781.2)	15	4267.4 (1465.7)	0.424
Eosinophils	59	57.7 (79.3)	15	30.2 (48.5)	0.233
Platelets	59	161498 (47357)	15	134580 (44249.9)	0.108
Alkaline phosphatase	49	77.3 (35.8)	7	75.8 (36.7)	0.790
Fibrinogen	21	505.0 (150.0)	5	562.8 (136.6)	0.224
Cortisol	44	19.05 (11.6)	5	23.3 (15.6)	0.357
Gamma-glutamyltransferase	50	77.5 (77.2)	9	97.6 (64.2)	0.177
Zinc	38	83.4 (15.1)	6	74.4 (20.6)	0.514
Prothrombin time	44	13.9 (3.7)	6	13.4 (0.59)	0.738
Albumin	41	3.7 (0.48)	6	3.8 (0.45)	0.697
Glycemia	47	126.4 (74.5)	8	138.1 (59.9)	0.242
ACE	42	36.1 (17.6)	6	35.3 (14.8)	0.999
pO2	55	73.3 (13.4)	14	79.8 (22.8)	0.687
CD8 cell count	15	382.3 (147.7)	3	265.3 (105.5)	0.250

SD: standard deviation; *n* (%); *p* values were calculated by the Mann–Whitney *U* test, *χ*2 test, or Fisher's exact test. CRP: C-reactive protein; LDH: lactate dehydrogenase; WBC count: white blood cell count; NT-proBNP: N-terminal prohormone of brain natriuretic peptide; ACE: angiotensin-converting enzyme: pO_2_: partial pressure of oxygen.

**Table 4:** Medications taken before admission and chest CT severity at admission compared to the patient's evolution to the ICU.

	Hosp_Intern (*n* = 59)	ICU (*n* = 15)	*p* value
*Used medications*			
Ceftriaxone	17 (23.0)	7 (9.5)	0.223
Hydroxychloroquine	34 (45.9)	6 (8.1)	0.221
Azithromycin	52 (70.3)	14 (18.9)	0.563
Ivermectin	27 (36.5)	11 (14.9)	0.056
Enoxaparin	25 (33.8)	8 (10.8)	0.446
Chlorpromazin	16 (21.6)	6 (8.1)	0.355

*Chest CT findings*			
Bilateral ground-glass opacity	43 (58.9)	15 (20.5)	0.030
Unilateral ground-glass opacity	8 (11.0)	0 (0.0)	
*Chest CT gravity*			
Involvement 25–50%	12 (16.4)	4 (5.5)	0.027
Involvement > 50%	1 (1.4)	3 (4.1)	
Pleural effusion	1 (1.4)	2 (2.7)	

*Chest CT nongravity*			
Clear	6 (8.2)	0 (0.0)	
Involvement < 25%	38 (52.1)	6 (8.2)	
Nodules	14 (19.2)	0 (0.0)	
Length of stay (days)	6.2 (±SD: 2.7)	10.7 (±SD: 8.7)	<0.001

*n* (%); *p* values were calculated by the Mann–Whitney *U* test, *χ*2 test, or Fisher's exact test.

## Data Availability

The data used to support the findings of this study are restricted by the Research Ethics Committee (Opinion No. 4.080.157, Santa Casa de Misericordia de Juiz de Fora/MG) in order to protect patient privacy. Data are available from Víctor de Oliveira Costa (e-mail: victordeoliveiracosta82@gmail.com) for researchers who meet the criteria for access to confidential data.
